# A Mechanistic Framework for Somatic Mosaicism in Autoinflammatory Diseases Beyond Mendelian Inheritance

**DOI:** 10.3390/ijms27198554

**Published:** 2026-09-24

**Authors:** Anoushka Krishnakumar, Elisabetta Tabolacci, Donato Rigante

**Affiliations:** 1 Department of Life Sciences and Public Health, Fondazione Policlinico Universitario A. Gemelli IRCCS, Largo A. Gemelli 8, 00168 Rome, Italy; anoushka.krishnakumar01@icatt.it; 2 UOC Genetica Medica, Fondazione Policlinico Universitario Agostino Gemelli IRCCS, 00168 Rome, Italy; elisabetta.tabolacci@unicatt.it; 3 Università Cattolica Sacro Cuore, 00168 Rome, Italy

**Keywords:** autoinflammatory disease, autoinflammation, genetic analysis, somatic mutation, mosaicism, personalized medicine, innovative biotechnologies

## Abstract

Hereditary autoinflammatory diseases have traditionally been defined as disorders caused by germline mutations in innate immune pathway genes transmitted through Mendelian inheritance, an assumption challenged by the discovery that postzygotic somatic mosaicism can reproduce similar autoinflammatory phenotypes. Aim of the review is to synthesize the mechanistic evidence for somatic and gonadal mosaicism across the major monogenic autoinflammatory diseases and to reassess the adequacy of “hereditary” as a categorical descriptor for this disease class. A structured narrative review of PubMed/MEDLINE was conducted (2002–2026), screening 241 records for evidence of somatic mosaicism in *NLRP3*, *MEFV*, *TNFRSF1A*, *NOD2*, *NLRC4*, *UBA1*, and *MVK*, of which 35 met inclusion criteria for qualitative synthesis; rather than proceeding disease-by-disease, evidence was organized around the underlying mechanisms by which a minority mutant-cell population produces disease. Four mechanistic categories were identified: (1) dominant effect without clonal expansion, most extensively documented in *NLRP3*-mediated cryopyrin-associated periodic disease; (2) clonal growth advantage, which introduces lineage-restricted diagnostic blind spots and, when gonadal tissue is involved, has implications for genetic counseling risk; (3) “passenger” mechanism converging with age-related clonal hematopoiesis in late-onset disease; and (4) no germline counterpart at all for *UBA1*-driven VEXAS syndrome. The detection of variants depends heavily on sequencing depth and tissue/lineage sampling, but variant allele fraction does not consistently predict clinical severity. Mevalonate kinase deficiency, a recessively inherited autoinflammatory disorder, has no reported mosaic cases, a pattern attributed to its biallelic genetic architecture rather than under-ascertainment. “Hereditary”, which conflates transmission and cellular distribution, is no longer an adequate categorical descriptor for autoinflammatory diseases as a class, though it remains accurate for individual patients and specific variants.

## 1. Introduction

Genetically driven autoinflammatory diseases are defined by recurrent episodes of fever and systemic inflammation, often with localized multi-organ symptoms and elevated acute-phase reactants [1]: these disorders primarily result from inherited defects of innate immune regulation, impacting the response gating through pattern recognition receptors, which coordinate cell migration, phagocytosis, cytolytic activity, and release of cytokines to patrol and manage cell turnover and death in jeopardized tissues [2]. Beyond monogenic diseases, a distinct category of polygenic or multifactorial autoinflammatory diseases should also be considered, including Still’s and Behçet’s diseases, in which multiple genetic loci cooperate with environmental triggers to produce a pathology [1,2]. Familial Mediterranean fever (FMF) is the prototypic and most common monogenic autoinflammatory disease, widely observed in populations of Mediterranean ancestry [3], whereas tumor necrosis factor receptor-associated periodic syndrome (TRAPS) [4], mevalonate kinase deficiency (MKD, also named hyperimmunoglobulinemia D syndrome-HIDS) [5], cryopyrin-associated periodic syndrome (CAPS) [6], Blau syndrome [7], and *NLRC4*-associated autoinflammatory diseases [8] represent less frequent entities within the autoinflammation spectrum. These conditions have been traditionally defined as monogenic hereditary disorders transmitted through classical Mendelian inheritance via germline variants, documentable in every cell of an affected individual [9,10]: this paradigm was established through the discovery of the first disease-causing gene in an autoinflammatory condition: indeed, two independent consortia identified variants in *MEFV* as the cause of FMF in 1997 [11,12], and shortly thereafter variants in *NLRP3* were shown to cause the three-sided range of CAPS, from familial cold autoinflammatory syndrome and Muckle-Wells syndrome to neonatal-onset multisystem inflammatory disease [6,13], solidifying *autoinflammatory diseases* as a germline genetic category for nearly two decades.

This germline-centric framework has been fundamentally challenged by the recognition that postzygotic somatic variants and mosaicism can produce clinically indistinguishable autoinflammatory phenotypes: more precisely, a substantial proportion of patients with autoinflammatory diseases may lack identifiable pathogenic variants in known disease genes, which may reflect overlapping protean clinical presentation, atypical or adult-onset disease, incomplete genomic assessment, and the contribution of yet-unidentified genes or modifier mechanisms to disease phenotype. The systematic investigation of patients with characteristic pictures, but negative germline testing, revealed low-level mosaicism in numerous genes including *NLRP3*, *NLRC4*, and *TNFRSF1A*, often escaping detection by conventional sequencing approaches [14,15,16,17]. Specific variants appear to occur exclusively in a mosaic state, suggesting they may be embryonically lethal when present constitutionally but pathogenic when confined to a subset of cells [18]. Moreover, the 2020 description of VEXAS syndrome extended this further, demonstrating that myeloid-restricted somatic variants in *UBA1*, arising through clonal hematopoiesis (CH), can cause a distinct acquired autoinflammatory disorder with no germline counterpart [19].

This current evidence necessitates reconceptualizing what constitutes “hereditary” in autoinflammatory diseases: unlike germline variants, somatic ones arise post-fertilization, are typically not transmitted to offspring, and produce phenotypes shaped by timing of the mutational event, affected cell lineage, and clonal dynamics [15,17,20,21]. This complexity challenges diagnostic and genetic-counseling frameworks built for germline disorders, and failure to recognize mosaicism risks missed diagnoses and delayed access to effective cytokine-targeted therapies, which are aimed at modulating the immune system: this is done by either inhibiting harmful pro-inflammatory signals that drive chronic inflammation and tissue damage or by enhancing specific immune responses leading to hyperinflammation [14,22].

Prior reviews of somatic mosaicism in autoinflammatory diseases have largely proceeded disease-by-disease; instead, this review organizes the evidence by the underlying mechanisms through which a minority mutant-cell population produces disease, using individual pathologic evidence to populate each mechanistic category, in order to directly address whether “hereditary” remains an adequate descriptor for this disease category.

## 2. Materials and Methods

This is a narrative review, not a formally registered systematic review, and no protocol was prospectively registered given its scope and objective. A structured medical literature search was performed in PubMed/MEDLINE to ensure comprehensive and reproducible source identification, covering publications from 2002 to 2026 (until August 2026). This specific window was chosen to capture the literature addressing somatic mosaicism as a disease mechanism; foundational gene-discovery papers predating this period (the original *MEFV* and *NLRP3*-related disease descriptions) were incorporated separately as background context rather than drawn from the systematic search itself.

Search terms spanned two domains: mechanism-level vocabulary (“somatic mosaicism”, “postzygotic mutation”, “clonal hematopoiesis”, “variant allele fraction”) and disease/gene-level vocabulary (“autoinflammatory disease”, “familial Mediterranean fever”, “cryopyrin-associated periodic syndrome”, “tumor necrosis factor receptor-associated periodic syndrome”, “mevalonate kinase deficiency”, “Blau syndrome”, “Schnitzler syndrome”, “VEXAS syndrome”, “*MEFV*”, “*NLRP3*”, “*TNFRSF1A*”, “*NOD2*”, “*UBA1*”, “*NLRC4*”), combined through Boolean operators. Titles and abstracts were screened first, with full-text review applied to potentially eligible records. As is standard for narrative reviews, no formal quality-appraisal instrument was used, though source selection was guided consistently by methodological rigor and relevance to the review’s mechanistic framework.

Eligible publications included case reports and case series, cohort studies, mechanistic or single-cell/molecular studies, systematic reviews, and clinical guidelines that reported somatic mosaicism in a monogenic autoinflammatory disease gene, with sufficient details on variant allele fraction (VAF), tissue of detection, or clinical phenotype to be informative. Publications were excluded if they were non-English, addressed germline-only variants with no mosaicism component, or were conference abstracts lacking adequate methodological detail–the latter retained only in the rare instance where no peer-reviewed alternative existed.

A total of 241 records were identified, and 35 were ultimately included in the qualitative synthesis. This was combined with 10 additional foundational and classification references incorporated in the Introduction. Independent of the systematic search, the reference list totals 45. The full selection process is summarized in Figure 1. Extracted data from each included source comprised study design, gene/disease, mosaicism detection method, VAF where reported, and the proposed disease mechanism.

## 3. Somatic Variants and Mosaicism: A Mechanistic Framework

The classical model of hereditary autoinflammatory diseases assumes that a causative variant is present in ‘every’ cell of the body, having arisen in the germline and inherited according to the Mendelian principles [21]. This assumption underlies not only diagnostic testing, which has historically searched for a single variant present at roughly 50% allele frequency, but also the genetic counseling frameworks built around it, which treat a negative parental test as conclusive evidence against recurrence risk [21,22]. Somatic mosaicism disrupts this model at its foundation: a disease-causing mutation confined to a subset of cells can produce a phenotype indistinguishable from its germline counterpart, while not being readily captured by the assumptions and methods designed for constitutional disease [23].

### 3.1. Types of Mosaicism

Postzygotic variants give rise to genetically distinct cell populations within a single individual, a phenomenon broadly termed ‘mosaicism’. Three principal categories are distinguished by the tissue distribution of the variant [21].

*Germline* (or *gonadal*) *mosaicism* refers to a variant confined to the cells that give rise to gametes, such that the variant is absent from somatic tissue but can be transmitted to offspring. *Somatic mosaicism* describes a variant present in non-gonadal tissue, arising at any point from early embryogenesis to adulthood, and is not heritable. *Gonosomal mosaicism* denotes the combined presence of a variant in both gonadal and somatic lineages; the variant is detectable in the patient’s own tissue and is simultaneously transmissible to the next generation [21].

This taxonomy, established in the broader context of human genetic diseases, applies directly to hereditary autoinflammatory disorders, where somatic and gonosomal mosaicism have emerged as an under-recognized cause of both “mutation-negative” diseases and apparent de novo cases [14]. Of the three categories, gonosomal mosaicism carries the most direct implications for genetic counseling, since it complicates the assumption that a proven-negative parent poses no recurrence risk. In a study of 92 families in which an affected child carried an apparently de novo disease-causing variant, deep amplicon sequencing identified parental gonosomal mosaicism in 6 families (6.5%)–carriers who were phenotypically unaffected and would have been classified as true de novo cases under standard Sanger-based parental testing [23]. This finding reframes “de novo” as, in part, an artifact of detection sensitivity rather than a stable biological category. As shown in Table 1, gonosomal mosaicism is the category most likely to be missed by conventional parental testing while still carrying transmission risk.

### 3.2. Timing and Origin of Somatic Variants

Postzygotic variants can arise at any point along a continuous developmental timeline, and the timing of a mutational event is the principal determinant of its clinical consequences [21]. A mutation occurring at the earliest cleavage divisions of the zygote is propagated to a large fraction of all descendant cells, producing widespread high-VAF mosaicism across multiple tissues and organ systems (mimicking a germline condition). As development proceeds, progressively later variants are confined to smaller and more restricted cell lineages, yielding lower-VAF tissue-limited mosaicism that may escape detection in commonly sampled tissue, such as the peripheral blood. Sequencing of parent-offspring trios has shown that a substantial proportion of variants long assumed to be germline in origin are, indeed, postzygotic events misclassified as de novo, due to the limited sensitivity of standard sequencing approaches [24]. At the far end of this timeline lies variant acquisition in adulthood, a process that is not itself pathological but is a near-universal feature of aging tissue. In the hematopoietic system, somatic variants accumulate in stem cells over a lifetime, and a variant that confers a proliferative advantage can drive expansion of a single mutated clone to comprise a disproportionate share of circulating blood cells. This is a phenomenon termed CH [25], quite common in older adults, and most frequently involving epigenetic regulators such as DNMT3A and TET2, and occurring in the absence of any hematologic malignancy or overt disease [25]. Framed alongside the embryonic end of the mosaicism spectrum, CH illustrates that not all late-acquired mosaicism is a disease mechanism in its own right; it is at baseline a normal consequence of aging blood.

### 3.3. How a Minority Mutant Cell Population Causes Disease

A minority population of mutated cells can produce clinically significant disease through several distinct mechanisms, and the mechanism at play in a given disorder has direct implications for how that disease is detected, monitored, and counseled around [26]. In the simplest case, a variant exerts a dominant or gain-of-function effect on cellular behavior without the mutant cell population itself expanding in size [26]. Here, disease severity is thought to depend less on the proportion of mutated cells and more on the potency of the mutant protein’s effect within the tissue where it is expressed [26]. The degree of phenotypic manifestations in mosaic disease has been shown to be shaped substantially by selection acting on the mutant clone, rather than by mutant cell fraction alone [26]. A second mechanism involves clonal growth advantage, in which the variant itself confers a proliferative or a survival benefit to the cell in which it arises [26]. Over time, this advantage allows the mutant clone to expand disproportionately relative to unmutated cells, increasing the effective disease burden even though the variant originated in a single cell [26]. This mechanism is distinct from the first in that disease severity may track more closely with clone size, and clone size itself may change over the course of a patient’s life [26]. A third and mechanistically distinct pathway has been termed the “passenger” mechanism, in which a disease-causing variant is carried along within a cell population that is expanding for an unrelated reason–the variant itself confers no growth advantage, but the cell lineage it resides in is expanding regardless [27]. This dynamic has been illustrated in a case of sporadic early-onset Alzheimer’s disease, in which a somatic *PSEN1* variant was found to be clonally amplified in the cerebral cortex by chance rather than by a proliferative effect of the variant itself [27]. This distinction matters clinically, since a passenger mutation’s future trajectory is governed by the dynamics of the unrelated expanding clone rather than by the property of the variant in question [27]. These three mechanisms–dominant/gain-of-function effect without expansion, clonal growth advantage, and the passenger mechanism are not mutually exclusive categorizations of individual variants so much as they are distinct explanatory frameworks, and a single disease-causing variant may be best explained by different mechanisms depending on the tissue and clonal context in which it is found [26,27].

### 3.4. Variant Allele Fraction and Its Clinical Correlations

VAF refers to the proportion of sequencing reads at a given genomic position that carry the mutant allele and serves as the standard quantitative measure of mosaicism burden within a sampled tissue [26]. The concept of VAF emerged with the development of massively parallel sequencing technologies, which enabled quantitative assessment of the proportion of sequencing reads carrying a given variant and, importantly, the detection of low-frequency variants associated with somatic mosaicism. A VAF of 50% is generally taken to indicate a heterozygous germline variant present in essentially all cells, while lower VAFs are interpreted as evidence of a postzygotic mosaic origin, with the specific proportion reflecting the size of the mutant cell clone within the sampled tissue [21]. The relationship between VAF and clinical phenotype has not been consistent across studies or across genes. In at least one systematic analysis of somatic *NLRC4* variants, a correlation was identified between the degree of mosaicism and both age of disease onset and overall clinical severity, with patients carrying higher mosaicism levels presenting earlier and with more pronounced symptoms [28]. In contrast, case-level analyses of somatic NLRP3 variants have found no such relationship, with neither disease severity nor phenotypic presentation tracking with the proportion of mosaicism detected in blood, including instances in which a low mosaicism level nonetheless produced a fully severe phenotype [29]. This inconsistency suggests that the clinical significance of a given VAF may depend on gene- and disease-specific factors such as the tissue distribution of the mutant clone, biological potency of the variant itself, and the mechanism by which the minority cell population produces disease.

### 3.5. Detection: Why Mosaicism Was Missed, and How It Is Found Now

Sanger sequencing was the predominant sequencing method in clinical genetic diagnostics for several decades, particularly from the 1990s through the early 2010s, before the widespread adoption of next-generation sequencing (NGS). Its averaged both-allele signal could only reliably detect variants present in roughly 15–20% or more of a sample, so it shaped which cases of mosaicism could be recognized at all. Variants present below this threshold are generally indistinguishable from background noise: as a result, a substantial proportion of mosaic variants, particularly those present in a small minority of sampled cells, would have gone entirely undetected under standard clinical workflows for decades, with cases only reclassified from “mutation-negative” or “de novo germline” to mosaic once more sensitive methods became available [30]. Several approaches developed to close this gap, each addressing the limitation differently, are listed in Table 2. Subcloning circumvented Sanger’s averaging problem by isolating individual DNA molecules prior to sequencing, but at the cost of substantial labor, time, and limited scalability, making it impractical for routine clinical diagnostics [21]. Deep NGS addressed the same problem computationally, generating a digital rather than analog readout [30]. Digital polymerase chain reaction (PCR) pushed sensitivity further still, partitioning a sample into thousands of individual reaction droplets for near-absolute quantification [31]. More recently, long-read sequencing and RNA sequencing (RNA-seq) have emerged as complementary approaches for the characterization of mosaic variants, although their application remains largely confined to research settings. Long-read sequencing can facilitate the analysis of complex genomic regions, structural variants, and phasing, whereas RNA-seq can provide transcript-level evidence, including aberrant splicing and allele-specific expression, when the relevant gene is expressed in the tissue analyzed.

A separate but related shift has come from how testing is initiated in the first place. Traditional testing has generally been phenotype-first. Broad genotype-first sequencing approaches such as trio-based exome or genome sequencing performed for suspected genetic disease generally without a specific gene hypothesis have instead surfaced mosaic variants incidentally, including cases that would never have been tested for directly [30]. This shift in strategy, independent of any improvement in assay sensitivity, has itself been a significant contributor to the growing recognition of mosaicism as a disease mechanism. Detection is further complicated by tissue and cell-type sampling. Because clinical testing is overwhelmingly performed on peripheral blood, a mosaic variant confined to a different tissue or a specific hematopoietic lineage may be missed entirely regardless of assay sensitivity, and the same variant may be detected or missed depending on which tissue happens to be sampled [30]. This tissue-specific distribution is also influenced by differences in mutational processes and mutation rates between germline and somatic compartments. While germline mutation rates are relatively constrained at the organismal level, somatic mutations accumulate throughout development and adult life, and can vary substantially between tissues according to cell turnover, replication history, DNA repair capacity, and clonal selection. The developmental timing of a post-zygotic event therefore influences both the VAF and the number and identity of tissues or cell lineages in which the variant can be detected. Within autoinflammatory diseases specifically, this sampling problem has been directly documented, with cases of low-frequency mosaicism identified only through deliberate multi-tissue or lineage-specific sampling strategies rather than standard single-tissue testing [32]. Establishing appropriate VAF thresholds for distinguishing genuine postzygotic variants from sequencing artifacts has similarly required dedicated methodological work in this context.

## 4. Mechanistic Categories of Somatic Mosaicism in Autoinflammatory Diseases

This section highlights the underlying mechanisms by which a minority population of mutated cells produces a disease. Figure 2 schematically summarizes the patterns discussed herein.

### 4.1. Dominant Effect Without Clonal Expansion

The first and best-documented mechanism (Figure 2, panel 1) is one in which a mutant clone causes disease purely through the potency of the variant itself, without any increase in the size of the mutant population over time. This pattern accounts for one of the most consistent findings in the mosaicism literature: patients with a fully classic phenotype and a negative germline genetic test. Because Sanger sequencing’s 15–20% VAF floor cannot reliably detect a static minority clone, a real disease-causing variant confined to a small fraction of cells can be phenotypically indistinguishable from a germline variant while remaining invisible to conventional testing [30]. *NLRP3*-associated diseases (i.e., CAPS) are the condition in which this pattern has been most systematically characterized. Low-level *NLRP3* mosaicism has been repeatedly identified in patients with classic CAPS phenotypes and negative conventional sequencing across both pediatric and adult cohorts [14,22]. Comparing germline and somatic *NLRP3* cases directly, one study found that the two groups may overlap in phenotype, and that a mosaic variant can reproduce the ‘full’ clinical spectrum of its germline counterpart despite being confined to a subset of cells [18]. A 2025 single-center UK cohort study built specifically around this question found that somatic *NLRP3* mosaicism accounted for a substantial share of pediatric CAPS cases that were found *NLRP3*-negative on standard sequencing, confirming the pattern persists even under modern testing protocols rather than being an artifact of the pre-NGS era [32]. As clinicians increasingly recognize mosaicism as a standing possibility in mutation-negative disease rather than a diagnostic dead end, referral for deeper sequencing has itself become part of standard evaluation for suspected monogenic autoinflammatory diseases [33].

*MEFV*-related disorder or FMF offers a rarer example of the same mechanism outside CAPS. A case report described an adult who developed a classic FMF phenotype associated with a somatic *MEFV* variant, the c.1955G > A, p.(Arg652His). Deep sequencing of hematopoietic subsets was performed after standard testing came back negative [34]. Because this *MEFV* variant arose alongside an unrelated *JAK2*-driven clonal expansion, this case sits at the boundary between this mechanism and the passenger mechanism, thereby illustrating that a single mosaic variant does not always belong to just one category.

Schnitzler syndrome, a rare adult-onset autoinflammatory condition characterized by chronic urticaria-like rash and monoclonal gammopathy of undetermined significance, represents a boundary case for this mechanism, where the evidence has proven harder to confirm. A case series identified myeloid-restricted somatic *NLRP3* mosaicism in two patients lacking the typical monoclonal gammopathy, thereby proposing ‘mosaicism’ as a possible explanation for that atypical presentation [35]. A considerably larger cohort study subsequently failed to replicate this: no *NLRP3* or *UBA1* somatic variants were found across their full Schnitzler syndrome population [36]. The two studies together illustrate a real limit of the mosaicism framework–a mechanism demonstrated convincingly in a small index cohort does not automatically generalize, and Schnitzler syndrome remains a setting where the mutation-negative explanation is unresolved rather than confirmed.

### 4.2. Clonal Growth Advantage

The second mechanism is one in which the mutant clone does not merely persist but expands, gaining a proportionally larger share of the cell population over time (Figure 2, panel 2). Because clone size itself can change over the course of a patient’s life, this mechanism carries two consequences that the first does not: detectability can depend heavily on which cell lineage and which point in time is sampled, and when the expansion involves gonadal tissue, the variant can become transmissible to offspring despite the parent appearing clinically unaffected.

#### 4.2.1. Lineage-Restricted Expansion and Diagnostic Sampling

Clonal growth advantage can act unevenly across blood cell lineages, so the same variant may be detectable in one cell population and invisible in another. In CAPS, myeloid-restricted somatic *NLRP3* mosaicism has been described, with the variant substantially enriched in myeloid relative to lymphoid cells. This is a distribution that non-myeloid sampling, such as lymphocyte fractions or buccal swabs, would be prone to miss [15].

A comparable pattern has since been documented in TRAPS, the most frequent autosomal dominant autoinflammatory disorder, where a somatic *TNFRSF1A* variant was detected in B cells, neutrophils, and natural killer cells but was absent from monocytes, T cells, and hair root DNA in the same patient [37]. These two cases across diverse genes and different diseases point to the same underlying implication: standard diagnostic sampling of whole blood or buccal tissue can systematically miss a real disease-causing clone simply because that clone’s growth advantage happened to favor a lineage the sampled tissue underrepresents.

#### 4.2.2. Gonosomal Mosaicism and Transmission Risk

When a clone’s growth advantage extends into gonadal tissue alongside somatic tissue, the resulting gonosomal mosaicism carries a genetic counseling consequence, which is the recurrence risk in future offspring despite a phenotypically unaffected test-negative parent. Blau syndrome, an autoinflammatory disorder that primarily affects the skin, joints, and eyes, caused by peculiar *NOD2* variants, provides the clearest demonstration of this risk realized in practice: a father with a mild late-onset phenotype was found to carry gonosomal *NOD2* mosaicism after the same variant was identified in both of his daughters, who presented with the more classic and severe pediatric disease course–the first documented case of intrafamilial Blau syndrome recurrence traced to gonosomal mosaicism in an apparently unaffected parent [38].

A parallel case in TRAPS reinforces the same pattern: a novel *TNFRSF1A* deletion, confirmed as a de novo mosaic variant with no detectable trace in either parent’s peripheral blood, illustrates the other side of this risk. Trio-based deep sequencing is essential to detect gonosomal mosaicism and accurately estimate a recurrence risk, avoiding false reassurance from the absence of detectable parental involvement [17]. Together, these cases support the broader claim that gonosomal mosaicism is disproportionately likely to be missed by conventional parental testing because it produces a phenotypically normal, genotypically mosaic parent.

### 4.3. The Passenger Mechanism and Convergence with Clonal Hematopoiesis

The third mechanism differs from the first two in a specific way: the disease-causing mutation itself confers no growth advantage on the cell that carries it. Instead, the variant is carried along within a cell population that is expanding for an entirely unrelated reason (Figure 2, panel 3). This distinction matters clinically, because a passenger mutation’s future trajectory is governed by the dynamics of whatever is driving the unrelated expansion, not by any property of the variant in question. A single genetically confirmed case makes this mechanism concrete. In a patient with adult-onset autoinflammatory disease, exome sequencing identified both a pathogenic *NLRC4* variant and a myeloid driver variant in *TET2*. Single-cell genotyping also confirmed that both variants occurred within the same hematopoietic clone. Their VAFs rose in parallel over time, consistent with a single stem or progenitor cell in which *TET2* drove clonal expansion, while *NLRC4* rode along, producing progressive *NLRC4*-inflammasome-driven autoinflammation as an incidental consequence of an expansion it did not cause [39]. In this case, *TET2*, which is an epigenetic regulator and one of the most frequently mutated genes in age-related CH, functioned as the clonal driver; its *loss-of-function* conferred the proliferative advantage that expanded the hematopoietic clone. *NLRC4*, an inflammasome-sensor gene, on the other hand, worked as the passenger: its *gain-of-function* variant triggered excess interleukin (IL)-1β/IL-18 release and drove the autoinflammatory phenotype without conferring any growth advantage of its own.

A subsequent longitudinal study of CAPS patients extends this same logic from a single case into a broader pattern. Comparing early-onset and late-onset somatic *NLRP3* mosaicism, Kato et al. found that late-onset cases had rising myeloid-enriched VAFs. In one case, single-cell sequencing found *NLRP3* and *TET2* variants within the same cells, with both allele fractions rising together over three years. This is the same driver-passenger signature seen in the *NLRC4*/*TET2* case, now observed directly in *NLRP3*-related diseases [40]. Early-onset cases in the same cohort showed neither pattern: VAFs remained stable across tissues and over time, distinguishing them mechanistically from the late-onset *TET2*-associated subset despite sharing the same causal gene.

VEXAS syndrome, caused by acquired somatic *UBA1* variants located on the X chromosome, represents where this convergence becomes most consequential. *UBA1* variants in VEXAS arise through the same CH process implicated in the *NLRC4*/*TET2* and late-onset CAPS cases, but unlike a passenger mutation riding an unrelated driver, the *UBA1* variant itself confers the clonal advantage. Here, a single variant drives both the disease and clonal expansion [19]. Positioned alongside the passenger cases, VEXAS demonstrates that age-related CH is not a peripheral detail relevant to only one disease but a shared substrate capable of producing autoinflammation through at least two distinct genetic architectures: an unrelated driver mutation dragging a passenger disease-gene along with it or the disease-causing mutation itself acting as the driver. Both routes converge on the same clinical picture, which is a late-onset, myeloid-restricted, rising-VAF disease in an older adult, despite arising through mechanistically distinct paths. This highlights the fact that age-related CH should be considered as a potential explanation whenever late-onset autoinflammatory diseases present with a myeloid-skewed, progressively rising mosaic variant, regardless of which gene is ultimately implicated.

### 4.4. Entirely Somatic Disease with No Germline Counterpart

The fourth category departs from the previous three in a fundamental way. It does not modify how a hereditary disease presents in a subset of cells; it describes a disease with no germline form and no Mendelian transmission at all (Figure 2, panel 4). VEXAS syndrome is the paradigm. Somatic variants affecting a single canonical residue of *UBA1* (p.Met41) were identified as the cause of a severe adult-onset autoinflammatory disease presenting with fevers, cytopenia, vasculitis combined with dermatologic manifestations, arising exclusively through CH in hematopoietic stem cells rather than through any inherited variant [19]. No parental transmission has been documented, and no constitutional or embryonic form of the disease exists to compare it against. A germline *loss-of-function* variant at this *UBA1* residue would be expected to compromise a ubiquitin-activating enzyme essential to cellular protein homeostasis, plausibly incompatible with survival to birth if present constitutionally. Because *UBA1* is X-linked and escapes X-inactivation, a variant confined to the single active allele in males has an outsized functional impact: VEXAS syndrome is accordingly described almost exclusively in males, though females account for roughly 4% of reported cases, nearly all associated with acquired or constitutional monosomy X alongside a somatic *UBA1* variant on the remaining allele [41,42]. Notably, germline *UBA1* variants are recognized as the cause of an entirely unrelated disorder, X-linked infantile spinal muscular atrophy [20], underscoring that VEXAS is not simply an under-ascertained germline autoinflammatory syndrome waiting to be found but a phenotype that appears to exist only in its acquired form. VEXAS represents the same driver-and-passenger logic seen in the *NLRC4*/*TET2* and late-onset CAPS/*TET2* cases, but with both roles collapsed into a single gene: in those cases, one gene (*TET2*) drove clonal expansion while a different one (*NLRC4*) rode along and caused a disease; in VEXAS, the same *UBA1* variant does both jobs at once. Rather than an unrelated driver variant dragging a passenger disease gene along with it, the *UBA1* variant is simultaneously the clonal driver and the disease-causing lesion. This is what makes VEXAS syndrome mechanistically distinct rather than merely a more severe instance of the passenger mechanism: the disease and clonal advantage are the same event, not two independent ones converging by chance. VEXAS is the strongest single piece of evidence available.

Every other mechanism involves a mosaic variant explaining an atypical presentation of an otherwise-recognized hereditary disease. *NLRP3*, *MEFV*, *TNFRSF1A*, and *NOD2* all have well-established germline forms, and mosaicism in each case sits alongside that germline disease as an alternative route to the same phenotype. *UBA1*/VEXAS has no such counterpart to sit alongside: it is not a somatic phenocopy of a hereditary disease, but a disease category with no hereditary form at all, occurring in a gene not classically associated with autoinflammatory diseases before its 2020 description [19]. This is the case that most directly forces the question this review was organized around: whether “hereditary” remains an adequate descriptor for autoinflammatory diseases as a category, given that at least one of its members is not hereditary under a strict definition of the term. Table 3 lists the mechanistic categories of somatic mosaicism in autoinflammatory diseases.

## 5. The Exception of Mevalonate Kinase Deficiency

Every mechanism examined in Section 4 shares a structural precondition: the underlying disease is dominantly inherited, and a single mutant allele confined to a minority of cells is sufficient to reproduce the germline phenotype. MKD/HIDS departs from this pattern at the level of inheritance itself: it is caused by biallelic *loss-of-function* variants in *MVK* inherited recessively from both parents; the disease requires a substantial reduction in mevalonate kinase enzymatic activity rather than a single dominant mutant allele acting within an otherwise wild-type cell [2,3,5,43,44]. No confirmed case of somatic *MVK* mosaicism producing systemic MKD/HIDS has been reported to date. The closest approach in the literature is a conference abstract describing genuine somatic mosaicism (approximately 22% VAF) for a novel *MVK* variant in an adult referred for suspected MKD; however, the patient’s recurrent fevers were ultimately attributed to Behçet’s disease rather than MKD, and the mosaic variant’s contribution to the phenotype was not exactly established [45]. Two further patients in the same series carried heterozygous *MVK* variants of undetermined significance that did not meet the biallelic threshold required for a confirmed diagnosis of MKD [45]. The absence of a positive case is therefore not simply a gap in reporting: it is consistent with what the recessive *loss-of-function* architecture of the disease would predict.

Reproducing MKD mosaically would require a second somatic mutation to occur in the same cell that already carries an inherited heterozygous variant (“second-hit theory”): that compound event would then need to affect enough of the body’s cell mass to drop systemic enzymatic activity below the threshold needed to dysregulate IL-1 production. This is a far less probable sequence than a single dominant *gain-of-function* variant establishing itself in a minority clone, which is all that CAPS, TRAPS, Blau syndrome, or VEXAS syndrome require. A detection-based explanation cannot be excluded alongside the mechanistic one. Deep sequencing of hematopoietic subsets has been pursued deliberately in *NLRP3*-negative CAPS cohorts, specifically because mosaicism was already suspected as an explanation for mutation-negative diseases [22,32]. No comparable dedicated search appears to have been undertaken in MKD/HIDS cohorts. Given how few patients present with a phenotype suggestive of MKD and negative biallelic testing to begin with, the absence of a positive finding may reflect an under-explored question rather than a settled biological impossibility. In *NLRP3*-, *MEFV*-, *TNFRSF1A*-, and *NOD2*-related diseases, somatic mosaicism may explain mutation-negative or atypical disease presentations; MKD breaks that pattern. Here, the barrier is not rarity but genetic architecture itself. The recessive biallelic genetic configuration of MKD presents a distinct constraint to a simple mosaic model, because disease requires pathogenic alterations of both *MVK* alleles, whereas a mosaic variant confined to a subset of cells would not necessarily reproduce the biallelic *loss-of-function* across the relevant cellular compartment.

## 6. A Debate Around the ‘Hereditary’ Content of Autoinflammatory Diseases

The question posed at the outset of this review, whether “hereditary” still holds once somatic mosaicism is accounted for, does not resolve to a single *yes* or *no* but forms a gradient rather than a binary break. Figure 3 places the four mechanisms along a single axis running from strictly hereditary germline diseases to entirely acquired diseases, with MKD branching off that axis entirely rather than occupying a position on it. The near-germline end of that axis is where diagnostic miss rates have historically been highest. Standard Sanger sequencing’s 15–20% VAF detection floor meant low-level mosaicism was systematically missed. Patients with classic phenotypes but no detectable germline variants were routinely treated as diagnostic dead ends [14,22,30]. Deep NGS has since shown a somatic variant confined to a subset of cells that can reproduce the full clinical severity of its germline counterpart [18]. Detection also depends on which tissue is sampled because postzygotic variants can carry lineage-restricted growth advantages; standard whole-blood or buccal sampling can still return a false negative, as seen in CAPS and TRAPS, where variants were enriched in specific leukocyte subsets and missed elsewhere [15,22,30,37]. A negative multi-gene panel should not be the end of the workup when targeted lineage-specific sequencing remains untried. Further along the axis, toward clonal growth advantage, genetic counseling protocols face their sharpest revision. A mutation absent from parents’ standard blood tests was treated as carrying negligible recurrence risk; genomic studies now show a meaningful share of such de novo variants are actually inherited from a parent with low-level gonosomal mosaicism. This is found in up to 6.5% of apparent de novo primary immunodeficiency cases once deep sequencing is used [23,24]. The intrafamilial transmission of Blau syndrome from an asymptomatic mosaic father to his affected daughters, alongside a comparable gonosomal TRAPS case, illustrates this [17,38]. Gonosomal mosaicism is the most insidious category for counseling, because it hides behind a negative parental blood test while retaining full transmission capacity [21,38]. At the acquired end of the axis, the most significant reframing is the intersection of somatic autoinflammatory disease with age-related CH [25].

Autoinflammatory diseases were historically seen as a pediatric category; CH explains severe late-onset cases that do not fit that pattern. Age-related driver variants in genes like *TET2* can pull along co-occurring *NLRC4* or *NLRP3* variants, producing late-onset disease as a byproduct of aging blood rather than of the passenger mutation’s own properties [38,39]. VEXAS pushes this further still, with the somatic *UBA1* variant acting simultaneously as trigger and clonal driver [9]. Together, these cases justify considering CH in any older adult with undifferentiated myeloid-skewed autoinflammatory disease, independent of family history [19,25]. Importantly, a more systematic assessment of the contribution of mosaic variants across autoinflammatory diseases, including their potential association with differences in disease onset, severity, or clinical presentation, would further clarify their biological and clinical relevance and may have important implications for diagnostic strategies and genetic counseling. This framework explains dominantly inherited diseases such as those mediated by *NLRP3* and *TNFRSF1A*, where a single *gain-of-function* allele suffices on its own [18,34,37], but does not translate to recessive *loss-of-function* disease. No confirmed case of somatic mosaicism producing systemic MKD/HIDS has ever been reported, a predictable consequence of its architecture rather than a gap in ascertainment. Reproducing MKD mosaically would require a second somatic hit in the same cell as an inherited heterozygous variant, affecting enough cell mass to drop enzymatic activity below the threshold needed to dysregulate IL-1, which is a far less probable event than a single dominant mutation establishing itself in a minority clone [43,44,45]. Together, Section 4 and Section 5 show that “hereditary” was always doing two jobs: describing transmission (germline, Mendelian) and describing distribution (present in every cell). Somatic mosaicism decouples these, and VEXAS syndrome shows they can be decoupled entirely. A single definition can no longer cover this disease group, which now spans four categories: purely germline disease, germline-origin disease with variable distribution, acquired disease that structurally mimics germline disease, and disease with no germline form at all. “Hereditary” remains accurate for individual cases and for genes like *MVK*, which is involved in MKD, close to universally accurate. Although somatic and gonosomal mosaicism are increasingly recognized, most autoinflammatory diseases are still caused by germline variants, particularly in familial forms. Thus, the term “hereditary” is appropriate when referring to diseases for which a germline genetic basis accounts for the majority of cases, while mosaic forms should be recognized as an important exception that may modify inheritance and recurrence-risk considerations.

## 7. The Road Ahead

The traditional classification of autoinflammatory diseases as strictly germline hereditary disorders should be revisited: the recognition that postzygotic somatic variants can mimic, modify, or entirely bypass classical Mendelian inheritance necessitates a fundamental shift in how these diseases are conceptualized, diagnosed, and managed. By evaluating the evidence through the lens of clonal mechanism rather than individual disease pathology, this review has highlighted the relevant clinical implications of mosaicism and challenges the adequacy of the “hereditary” descriptor. That challenge is not uniform. The four mechanisms reported have traced a spectrum from mosaicism, which merely redistributes a familiar germline mutation, to VEXAS syndrome, a disease with no germline form at all, while MKD shows this erosion tracks genetic architecture rather than applying across autoinflammatory diseases indiscriminately. Substantial gaps remain: evidence density is uneven across genes, VAF-severity relationships are inconsistent, and whether MKD’s resistance reflects true biological impossibility or simply a lack of dedicated screening is still unsolved.

Future work should prioritize standardized deep sequencing as first-line workup for phenotype-positive/genotype-negative diseases. It should also include longitudinal VAF studies to test whether clone size predicts disease trajectory, and mechanistic studies extending CH convergence findings beyond the current small number of confirmed cases. Ultimately, how well diagnosis, counselling, and treatment keep pace with autoinflammatory diseases will depend on how fully this mosaic, only partly hereditary, picture is recognized in practice.

## Figures and Tables

**Figure 1:**
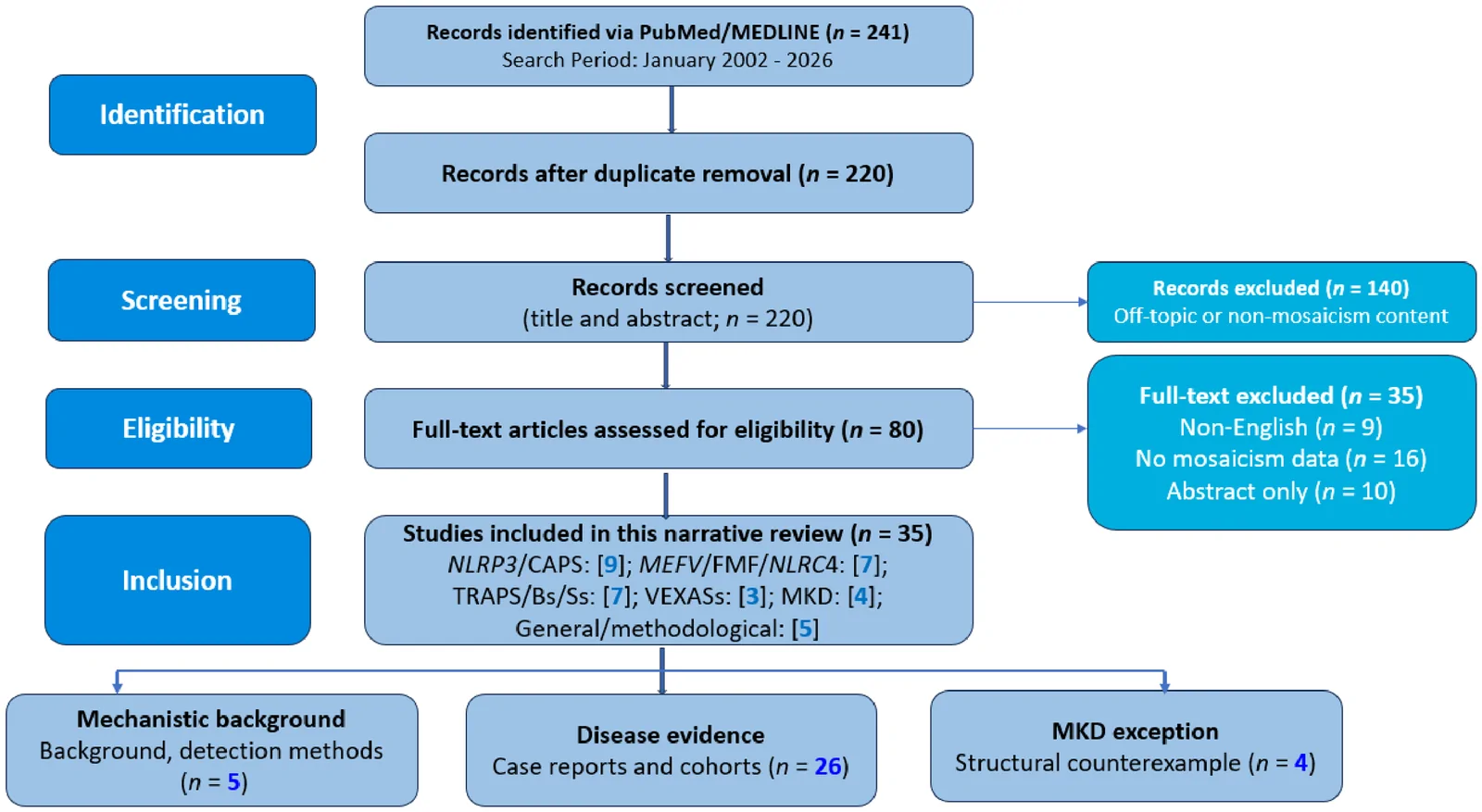
Flow diagram detailing the narrative review process used in our research of papers dedicated to the aim of this review. **Abbreviations:** CAPS: cryopyrin-associated periodic syndrome; TRAPS: tumor necrosis factor receptor-associated periodic syndrome; Bs: Blau syndrome; Ss: Schnitzler syndrome; VEXASs: vacuoles, E1 enzyme, X-linked, autoinflammatory, somatic syndrome; MKD: mevalonate kinase deficiency.

**Figure 2:**
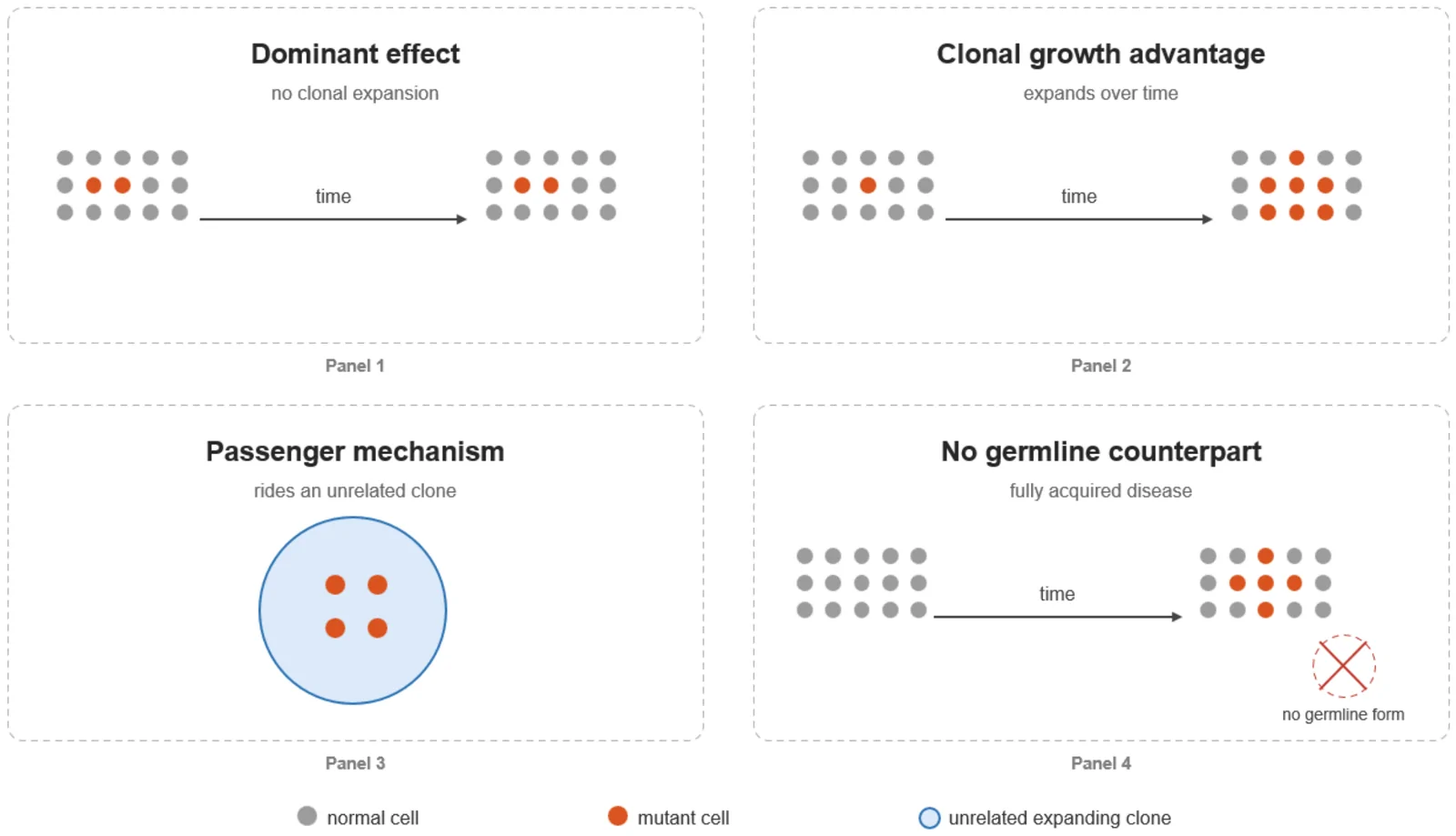
Four mechanistic patterns by which a minority mutant-cell population causes disease: (1) a dominant or *gain-of-function* effect exerted by the mutant clone without any expansion in its size; (2) a growth advantage that increases the mutant clone’s proportional representation over time; (3) a passenger mechanism in which the variant is carried along within a cell population expanding for an unrelated reason; (4) a disease that is entirely somatic in origin, with no corresponding germline form.

**Figure 3:**
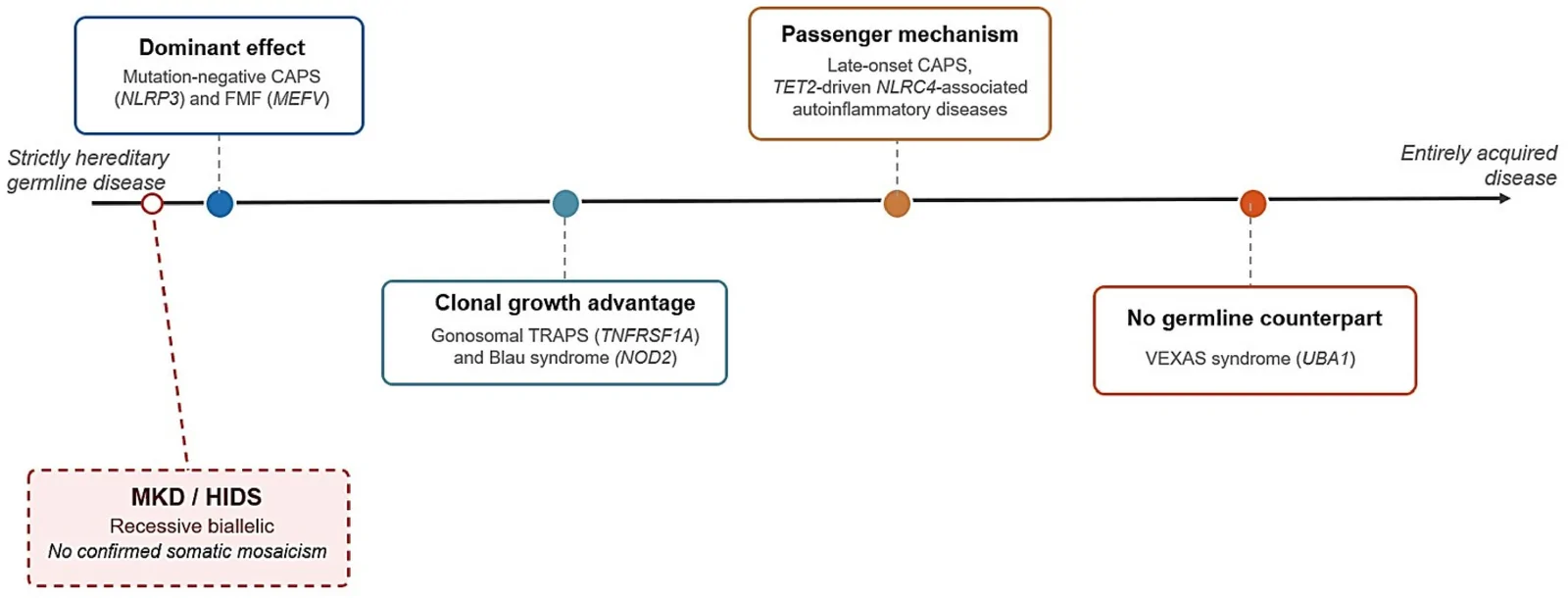
Mechanistic categories placed along an axis from strictly hereditary to entirely acquired autoinflammatory diseases. Mevalonate kinase deficiency branches off the axis since no confirmed case of somatic mosaicism has been reported in this recessive biallelic *loss-of-function* disease. *Abbreviations*: HIDS, hyperimmunoglobulinemia D syndrome; MKD, mevalonate kinase deficiency; VEXAS syndrome, vacuoles, E1 enzyme, X-linked, autoinflammatory, somatic syndrome.

**Table 1:** Different types of mosaicism with their subsequent clinical implications.

*Type*	*Tissue Distribution*	*Heritable*	*Detectable via Standard Parental Testing*	*Genetic Counseling Implication*
Germline (gonadal)	Confined to gamete-forming cells; absent from somatic tissue	Yes	No, parent is phenotypically and genotypically normal in blood/somatic (buccal) samples	Recurrence risk in future offspring despite an apparently unaffected “test-negative” parent
Somatic	Present in non-gonadal tissue; distribution depends on timing of the postzygotic mutation	No	Yes, if the sampled tissue carries the variant at sufficient VAF	None for offspring; risk is confined to the mosaic individual and depends on which tissues/organs are affected
Gonosomal	Present in both gonadal and somatic lineages	Yes	Partially, may be detectable in blood/somatic cells if VAF is sufficient, but standard testing can still miss low-level cases	Combines both risks: the carrier may have mild/subclinical disease and can transmit the variant to offspring

**Abbreviations:** VAF: variant allele fraction.

**Table 2:** Detection methods for somatic mosaicism in autoinflammatory diseases by sensitivity and specific approaches.

*Method*	*Approximate VAF Detection Floor/Sensitivity*	*Mechanism*	*Key Limitation*
Sanger Sequencing	15–20%	Bulk analog signal, both alleles averaged	Intensive to low-level mosaicism
Subcloning	Below Sanger floor	Individual DNA molecules are isolated and sequenced separately	Labor-intensive—each DNA molecule must be isolated and sequenced individually rather than in bulk, so throughput is low
Deep NGS	1–10%	Digital, read-by-read variant count	Cost/depth trade-off
Digital PCR	<0.1%	Sample partitioned into thousands of droplets	Requires known target variant; not exploratory
Genotype-first exome/genome sequencing	Depends on sequencing depth used	Broad sequencing without prior gene hypothesis	May still miss tissue-restricted variants
Long-read sequencing	Platform- and depth-dependent	Long individual reads enable phasing and characterization of complex genomic regions and structural variants	Currently more commonly used in research settings; sensitivity to low-level mosaicism depends on coverage and platform
RNA sequencing (RNA-seq)	Not directly applicable	Transcript-level analysis can provide evidence of expressed variants, aberrant splicing, and allele-specific expression	Requires gene expression in the sampled tissue; mainly a complementary/research approach

*Abbreviations*: NGS: next-generation sequencing; PCR: polymerase chain reaction; VAF: variant allele fraction.

**Table 3:** Summary of mechanistic categories of somatic mosaicism in autoinflammatory diseases.

*Mechanism (Section)*	*Representative Gene(s)/Disease(s)*	*Key Evidence*	*VAF Pattern*	*Clinical/Counseling Implication*
Dominant effect without clonal expansion (Section 4.1)	*NLRP3*/CAPS (repeated cohort evidence); *MEFV*/FMF (single case report); Schnitzler syndrome	Repeated identification of low-level *NLRP3* mosaicism in mutation-negative CAPS across pediatric and adult cohorts [14,22,32]; comparable germline/somatic phenotypes [18]; single *MEFV* case [34]; conflicting Schnitzler findings [35,36]	Low, static; does not correlate with severity	Deep sequencing should be considered in phenotype-positive/genotype-negative patients, as a negative Sanger result does not exclude the diagnosis
Clonal growth advantage (Section 4.2)	*NLRP3*/CAPS (myeloid-restricted); TRAPS (lineage-restricted; gonosomal); Blau syndrome (gonosomal)	Myeloid-restricted *NLRP3* mosaicism undetectable in non-myeloid tissue [15]; TRAPS variant present in some leukocyte lineages but not others [37]; Blau syndrome: documented parent-to-child transmission via gonosomal mosaicism [38]; TRAPS: de novo gonosomal case illustrating detection-sensitivity risk [17]	Rising over time and/or lineage-skewed	Tissue/lineage choice affects detection; gonosomal cases carry real recurrence risk in offspring of a phenotypically normal, test-negative parent
Passenger mechanism (Section 4.3)	*NLRC4*/*TET2*; late-onset CAPS/*TET2*; converges conceptually with VEXAS syndrome	Single-cell confirmation of *NLRC4* and *TET2* variants in the same clone with parallel VAF rise [39]; equivalent *NLRP3*/*TET2* co-occurrence in late-onset CAPS [40]	Rising in parallel with an unrelated clonal hematopoiesis driver	Consider clonal hematopoiesis workup (e.g., *TET2*) in late-onset, myeloid-skewed mosaic autoinflammatory disease regardless of which gene is implicated
No germline counterpart (Section 4.4)	*UBA1*/VEXAS syndrome	Paradigm case: X-linked, clonal hematopoiesis-driven, no constitutional or inherited form described [19]	High, myeloid-restricted, clonal hematopoiesis-driven	No offspring recurrence risk to counsel on; it should be considered in adult-onset undifferentiated inflammatory disease with cytopenia, independent of any germline hypothesis

*Abbreviations*: CAPS: cryopyrin-associated periodic syndrome; FMF: familial Mediterranean fever; VEXAS: vacuoles, E1 enzyme, X-linked, autoinflammatory and somatic.

## Data Availability

No datasets were generated during the present study.

## Abbreviations

The following abbreviations are used in this manuscript:

FMF	familial Mediterranean fever
TRAPS	tumor necrosis factor receptor-associated periodic syndrome
MKD	mevalonate kinase deficiency
HIDS	hyperimmunoglobulinemia D syndrome
CAPS	cryopyrin-associated periodic syndrome
VEXAS	vacuoles, E1 enzyme, X-linked, autoinflammatory, somatic
VAF	variant allele fraction
CH	clonal hematopoiesis
NGS	next-generation sequencing
PCR	polymerase chain reaction
